# Acute social defeat during adolescence promotes long-lasting aggression through activation of the medial amygdala

**DOI:** 10.3389/fnins.2024.1433993

**Published:** 2024-07-10

**Authors:** Nooshin Mojahed, Magdalene Adjei, Elana Qasem, Sophia Aaflaq, Temitope Adu, Jessica T. Jacobs, Ben D. Richardson, Jacob C. Nordman

**Affiliations:** ^1^Department of Biomedical Sciences, Southern Illinois University School of Medicine, Carbondale, IL, United States; ^2^Department of Pharmacology, Southern Illinois University School of Medicine, Springfield, IL, United States

**Keywords:** social defeat, traumatic stress, aggression, medial amygdala, chemogenetics, ventromedial hypothalamus

## Abstract

Traumatic stress, particularly during critical developmental periods such as adolescence, has been strongly linked to an increased propensity and severity of aggression. Existing literature underscores that being a victim of abuse can exacerbate aggressive behaviors, with the amygdala playing a pivotal role in mediating these effects. Historically, animal models have demonstrated that traumatic stressors can increase attack behavior, implicating various amygdala nuclei. Building on this foundation, our previous work has highlighted how traumatic stress invokes long-lasting aggression via an excitatory pathway within the posterior ventral medial amygdala (MeApv). In the current study, we sought to further delineate this mechanism by examining the effects of acute social defeat during adolescence on aggressive behaviors and neural activation in mice. Using a common social defeat paradigm, we first established that acute social defeat during late adolescence indeed promotes long-lasting aggression, measured as attack behavior 7 days after the defeat session. Immunolabeling with c-Fos demonstrated that acute social defeat activates the MeApv and ventrolateral aspect of the ventromedial hypothalamus (VmHvl), consistent with our previous studies that used foot shock as an acute stressor. Finally, chemogenetically inhibiting excitatory MeApv neurons during social defeat significantly mitigated the aggression increase without affecting non-aggressive social behavior. These results strongly suggest that the MeApv plays a critical role in the onset of aggression following traumatic social experience, and offer the MeA as a potential target for therapeutic interventions.

## Highlights

- Acute social defeat during adolescence promotes long-lasting aggression.
- Acute social defeat activates the posterior ventral medial amygdala (MeApv) and ventrolateral ventromedial hypothalamus (VmHvl).
- Excitatory MeApv neurons mediate acute social defeat-induced aggression.

## 1 Introduction

Both bullying and child abuse are potent stressors that can deeply affect brain structure and function, especially in regions associated with aggression, impulse control, and emotion regulation (Martin et al., 2006; Hart and Rubia, 2012). Significant evidence from neurobiological studies indicates that individuals who have endured severe stress or trauma during childhood often exhibit physiological, structural, and functional alterations in the amygdala, a critical brain region involved in emotional responses. These changes can lead to heightened aggression and difficulties in impulse management, potentially contributing to a perpetuation of violence across the lifespan—a phenomenon often described as the “cycle of violence” (Widom, 1989).

The amygdala plays a crucial role in the emotional processing of threats and fear, influencing aggressive behaviors that are central to the cycle of violence (Davis and Whalen, 2001). In those who have experienced early life abuse or severe stress, the amygdala may become hyperactive or overly sensitive to perceived threats, leading to enduring neural alterations (McCrory et al., 2010). This hyperactivity is believed to contribute to increased aggression, often thought to be a defense mechanism (Coccaro et al., 2007).

Among the various subregions of the amygdala, the medial amygdala (MeA) is particularly significant in the context of violent aggression (Nelson, 2006; Nelson and Trainor, 2007; Hong et al., 2014; Nordman J. et al., 2020; Nordman J. C. et al., 2020). It has been successfully targeted in neurosurgical interventions aimed at treating intractable, escalated aggression (Mpakopoulou et al., 2008). Building on this foundation, our recent research demonstrated that traumatic stress, induced by non-contingent foot shock, significantly enhances aggression through the strengthening of synaptic connections between the posterior ventral segment of the MeA (MeApv) and the ventrolateral segment of the ventromedial hypothalamus (VmHvl) (Nordman J. et al., 2020; Nordman J. C. et al., 2020; Nordman et al., 2022; Bartsch et al., 2023b). Potentiation of these MeApv-VmHvl synapses results in excessive and recurrent attack behavior that persists into adulthood. Furthermore, we found that optogenetically weakening these synapses after exposure to traumatic stress can reduce the escalation of aggression, whereas strengthening them can mimic the effects of traumatic stress on aggression.

While non-contingent foot shock effectively induces excessive aggression in animal models, its relevance to the complex and varied nature of human stressors, both social and physical, is limited. The specific and isolated nature of this stressor does not fully encapsulate the breadth of human traumatic experiences. To address this limitation and enhance the ethological validity of our model, in the current study, we replaced acute foot shock with acute social defeat (SD), where the subject animal is attacked by a larger more aggressive conspecific, in this case, a mouse strain known as CD1 (Golden et al., 2011). This method better mirrors the social stressors that humans commonly encounter and captures the complexity of these interactions more accurately.

Our findings reveal that SD not only promotes long-lasting aggression but also leads to significant activation of the MeApv and VmHvl. To determine the role of the MeApv in SD-induced aggression, we chemogenetically inhibited the excitatory neurons within the MeApv, the MeApv neuron type we previously identified as responsible for foot shock-induced aggression (Nordman J. et al., 2020; Nordman J. C. et al., 2020). Our manipulations resulted in a marked suppression of aggression without affecting non-aggressive social behavior. These results strongly suggest that the aggression increase after SD is driven by activation of the MeApv.

These findings advance our understanding of the neural mechanisms underlying aggression and offer valuable insights into potential targets for therapeutic interventions.

## 2 Materials and methods

### 2.1 Animals

All animal protocols were approved by the Animal Care and Use Committee of Southern Illinois University School of Medicine. All C57BL/6 mice used for histochemical and behavioral experiments were purchased from Charles River Laboratories. All C57BL/6 mice used for electrophysiology experiments were bred in-house. Both groups were housed under a reverse 12-h light cycle with *ad libitum* access to water and food. Starting at 3–4 weeks of age, mice were socially isolated for 4 weeks before testing. Surgical procedures, outlined below, were performed at 4–5 weeks of age and then the mice were socially isolated for an additional 3 weeks. All social defeat and aggression tests were conducted at the same age 7–8 :weeks for defeat and 8–9 weeks for aggression testing. Male mice were used for all experiments due to sex-specific effects of the MeA on aggression (Unger et al., 2015).

### 2.2 Acute social defeat-induced aggression

After 4 weeks of social isolation in a reverse light cycle, surgical animals were transferred from their housing room to a darkened behavior room and left alone to acclimate for 1 h prior to stress induction. They were then placed into the home cage of a larger, more aggressive CD1 mouse for 10 min. This technique is widely used due to the reliability and intensity of CD1 attacks against an intruder (Golden et al., 2011). After the 10-min defeat period, the C57 mice were removed and returned to their home cage. Only mice that were attacked within 1 min of being introduced to the CD1 were used for further testing (Fujii et al., 2019). If excessive tissue damage occurred, the test was prematurely terminated. Control mice were left in an empty cage for 10 min before returning them to their home cage.

Mice were then divided into two groups. One group was euthanized 30 min after SD to be used for immunohistochemistry experiments, described below. The other group was used for aggression testing 7 days later. These mice were again transferred from their housing room to a darkened behavior room and left alone to acclimate for 1 h prior to aggression testing. For aggression testing, the mice were placed in a novel, high walled cage and left to acclimate for an additional 20 min. Three to four-week-old, group-housed target males (conspecific) were introduced into the same cage with the experimental mice, and the two were allowed to freely interact for 10 min. Animal behavior was captured with a video camera. If excessive tissue damage occurred, the test was prematurely terminated and not analyzed. Mice that exhibited total attack time > 40% of the test period were eliminated from further analysis (Nordman J. et al., 2020; Nordman J. C. et al., 2020). Videos of behavior tests were reviewed and hand scored by a researcher blind to the experimental conditions. Aggressive behaviors were defined as bites to the rear, bites to the front/face, chasing, and wrestling (Blanchard and Blanchard, 1977; Koolhaas et al., 2013; Nordman and Li, 2020; Nordman J. et al., 2020; Nordman J. C. et al., 2020). Non-aggressive social behavior was defined as anogenital sniffing, investigation, and flank rubbing. Non-social behavior such as paw-licking, facial and body grooming, exploring, or when non-ambulatory was designated as other.

### 2.3 Foot shock

8–9-week-old male C57BL/6 mice were transferred from their housing room to a behavior room shielded from light and then allowed to acclimate for 1 h before being placed into a white light illuminated fear conditioning chamber within a sound-attenuating cubicle (Med Associates). After a 3 min exploration period, three electric shocks (0.7 mA, 1 s in duration) were administered through an electrified grate at random intervals of 240–480 s over 15 min. Mice were then returned to their home cage for 30 min and then tested for aggression or euthanized for immunohistochemistry experiments.

### 2.4 Immunohistochemistry

Mice were transcardially perfused with 4% paraformaldehyde in phosphate buffered saline solution (PBS) 30 min after foot shock. Brains were removed and post-fixed at 4°C overnight, then cryoprotected overnight in 15% sucrose in PBS followed by 30% sucrose in PBS. Brains were cut into 30-μm thick sections using a cryostat (Leica CM3050-S) and then stored in PBS as floating sections for immunohistochemistry or for confirming the location of viral injection. For immunohistochemistry experiments, free floating brain sections were blocked with 10% goat serum and 1% bovine serum albumin in PBS-T (PBS with 0.03% Triton X-100) for 2 h at room temperature. Sections were then incubated in c-Fos antibody (1:2,000, Abcam, #ab190289) overnight at 4°C, followed by incubation with a 488 Alexa Fluor secondary antibody (1:200, ThermoFisher, #A-11034) or 555 Alexa Fluor secondary antibody (ThermoFisher, #A-21428) for 1 h at room temperature. Sections were then mounted to slides with Vectashield HardSet Antifade Mounting Medium (Vector Laboratories) containing DAPI.

### 2.5 Image acquisition and image analysis

Brain slices were imaged with a fluorescent microscope (Evos) with a 10x (NA 0.3) and 20x (NA 0.4) objective. c-Fos positive cells were identified using the “Analyze Particles” function of ImageJ and validated as cells by their overlap with DAPI. Cells co-labeled for DAPI, mCherry, and/or c-Fos were manually counted by a researcher blind to the experimental conditions.

### 2.6 Surgical procedures

Four to five–week-old C57BL/6 male mice were anaesthetized with isoflurane (3% for induction and 1–2% for maintenance) and then placed onto a stereotaxic frame (David Kopf Instruments). A unilateral (for electrophysiology only) or bilateral (for histochemical and behavioral experiments) craniotomy was made and 250–300 nL of a viral suspension was injected into each MeApv (AP = −1.5 mm, ML = ±2.1, DV = −5.25 mm) using a 5–10 μL gas-tight Hamilton Syringe coupled to a 33-gauge stainless steel needle or capillary glass pipette with a 20 μm diameter opening at a rate of 25–30 nL/min. After injection, the needle was left in place for an additional 10 min and then slowly withdrawn. The adeno-associated viruses (AAV) used were pAAV2-CaMKIIα-hM4D(Gi)-mCherry (hM4D(Gi), Addgene #50477, 1.0–2.1 × 10^13^ vg/mL) and pAAV9-CaMKIIα-mCherry (mCherry, Addgene #114469, 2.1 × 10^13^ vg/mL). Skin was sealed using Vetbond or surgical staples. Ketoprofen or meloxicam were administered for 3 days post-surgery. Mice were allowed to recover for 2–4 weeks.

### 2.7 Electrophysiology

Three to four weeks after receiving a pAAV2-CaMKIIα-hM4D(Gi)-mCherry unilateral injection into the MeApv, mice were anesthetized with 4% isoflurane and transcardially perfused with 20–25 mL of ice-cold *N*-methyl-D-glucamine (NMDG)-based slicing solution (see below) and then decapitated. The brain was then rapidly extracted, embedded in agar, and sliced (300 μm thick) with a Compresstome (Precisionary Instruments) vibrating tissue slicer, all in ice-cold NMDG-based slicing solution. Slices were then transferred to a holding chamber containing 50 mL of warmed (35°C) NMDG-slicing solution, after which the NaCl concentration was systematically increased at 5 min intervals over 25 min (Ting et al., 2018). After raising the NaCl concentration, slices were transferred to a HEPES-based holding solution (described below) and maintained at 35°C for 30 min before lowering the temperature to room temperature where they remained until being transferred to the recording chamber individually over the subsequent 6–7 h. The NMDG-based slicing solution contained (in mM): 92 NMDG, 92 HCl, 2.5 KCl, 1.2 NaH_2_PO_4_, 30 NaHCO_3_, 10 MgCl_2_, 0.5 CaCl_2_, 20 HEPES, 25 glucose, 2 ascorbic acid, 2 thiourea, and 3 sodium pyruvate; and the HEPES-based holding solution contained (in mM): 92 NaCl, 2.5 KCl, 1.2 NaH_2_PO_4_, 40 NaHCO_3_, 2 MgCl_2_, 2 CaCl_2_, 20 HEPES, 25 glucose, 2 ascorbic acid, 2 thiourea, 3 sodium pyruvate, and 3 myo-inositol, both solutions were continuously perfused with 95% O_2_/5% CO_2_, had pH 7.3–7.4 and osmolarity of 300–310 mOsm.

For whole cell electrophysiology recordings of MeApv neurons, slices were transferred to a recording chamber and continuously perfused at 3–5 mL/min with artificial cerebrospinal fluid (ACSF) at 32–34°C, which contained (in mM): 125 NaCl, 2.5 KCl, 1 NaH_2_PO_4_, 26 NaHCO_3_, 2 MgCl_2_, 2 CaCl_2_, 20 glucose, 2 ascorbic acid, and 3 myo-inositol, that was continuously perfused with 95% O_2_/5% CO_2_ and had an osmolarity of 310–320 mOsm. MeApv neuronal cell bodies were visualized with a 60x water immersion objective on an Olympus BX51WI microscope with infrared differential interference contrast imaging. Only neurons within the MeApv that expressed mCherry visualized with a halogen lamp and an appropriate filter set (530–550 nm excitation/590-nm emission) were used for whole cell patch-clamp recordings. Patch clamp electrodes were prepared from borosilicate capillary glass (1.5 mm OD/0.86 mm ID) with a Sutter P1000 Micropipette Puller to have a resistance of 3–5 MΩ when filled with an internal solution containing (in mM): 139.6 K-Gluconate, 0.4 KCl, 4 NaCl, 0.5 CaCl2, 10 HEPES, 5 EGTA, 4 Mg-ATP, and 0.5 Na-GTP with a pH of 7.2–7.3 and osmolarity adjusted with KOH to 285-290 mOsm. Current clamp and voltage clamp recordings were acquired with a MultiClamp 700B amplifier and digitized at 20 kHz (10 kHz low pass filter) with a Digidata 1440 (Molecular Devices).

For all recordings, an initial high resistance gigaseal was formed (>1GΩ), followed by negative pressure application to obtain the whole cell configuration, which was maintained for at least 5 min before data acquisition. After this 5 min initial period, a 5 mV voltage step was applied in voltage clamp mode to evaluate membrane capacitance and resistance along with series resistance. If the series resistance exceeded 30 MΩ (15.5 ± 1.1 MΩ) or changed by more than 30% over the duration of a recording the data were discarded. The membrane potential (not corrected for the liquid junction potential) was then recorded in current clamp mode for 2–3 min to establish the baseline value of the resting potential before applying clozapine-N-oxide (10 μM, CNO, Sigma) in the ACSF or marking a timepoint as the “vehicle” administration timepoint. Most neurons did not spontaneously generate action potentials, but for those that did, the peak (μ) of an all points histogram was used to establish the membrane potential over a time point. To determine the membrane potential, a 10 s period was averaged at the indicated intervals and analyzed relative to the time CNO or “vehicle” entered the bath reservoir.

### 2.8 Chemogenetic experiments

3 weeks after viral injection, surgical animals were transferred from their housing room to a darkened behavior room and left alone to acclimate for 1 h prior to stress induction. Mice were injected with 0.9% saline (vehicle) or 1 mg/kg clozapine N-oxide (CNO, Sigma) (Chang and Gean, 2019; Bartsch et al., 2023b) 30 min before SD or foot shock.

### 2.9 Statistical analysis

All data were presented as individual data points or expressed as mean ± SEM. GraphPad Prism software was used for statistical analysis. Student’s *t*-tests were used in Sections 3.1, 3.2, and 3.3 (as they passed normality) to compare the amount of aggressive and non-aggressive social behavior between attacked mice and controls (Section 3.1), the number of c-Fos + and DAPI+ cells between attacked mice and controls (Section 3.2), and the number of mCherry+ neurons that colocalized with c-Fos between CNO and vehicle-treated hM4D(Gi)-expressing mice (Section 3.3). For electrophysiological recordings of CNO or vehicle effects on MeApv neuronal resting membrane potential, paired *t*-tests were used to compare the membrane potential prior to and after CNO or vehicle within a cell, and a *t*-test was used to compare the degree of drug/vehicle-induced change in membrane potential between cells exposed to CNO or vehicle. For the chemogenetic behavioral experiments, two-way ANOVAs were used to compare aggressive and non-aggressive social behavior between groups that were transduced with hM4D(Gi) or mCherry control virus and received CNO or vehicle 30 min before SD. Tukey’s test was used for *post-hoc* multiple comparisons to identify groups that were significantly different. *p* < 0.05 was considered significant, and all tests were two-tailed.

## 3 Results

### 3.1 Acute social defeat during adolescence increases attack behavior

We began by assessing the impact of SD during adolescence on long-lasting aggression in C57 mice. We previously showed that traumatic stress during adolescence in the form of non-contingent foot shock could promote long-lasting aggression (Nordman J. et al., 2020; Nordman J. C. et al., 2020; Bartsch et al., 2023a,b). However, given the comorbidity of childhood physical abuse and violent aggression later in life (Widom, 1989; Caspi et al., 2002; Widom and Brzustowicz, 2006), and to simulate a more ethologically realistic stressful encounter to humans, for this study we elected to use SD as our traumatic stressor.

To initiate SD, we first subjected 3–4-week-old C57 mice to a period of social isolation for 4 weeks. This phase was intended to model chronic early life stress and potentially prime the animals to subsequent acute stressors. Importantly, we previously showed that social isolation alone does not increase aggression in mice if tested in a novel arena (Nordman et al., 2022; Bartsch et al., 2023a), as was used in this study.

**Figure 1:**
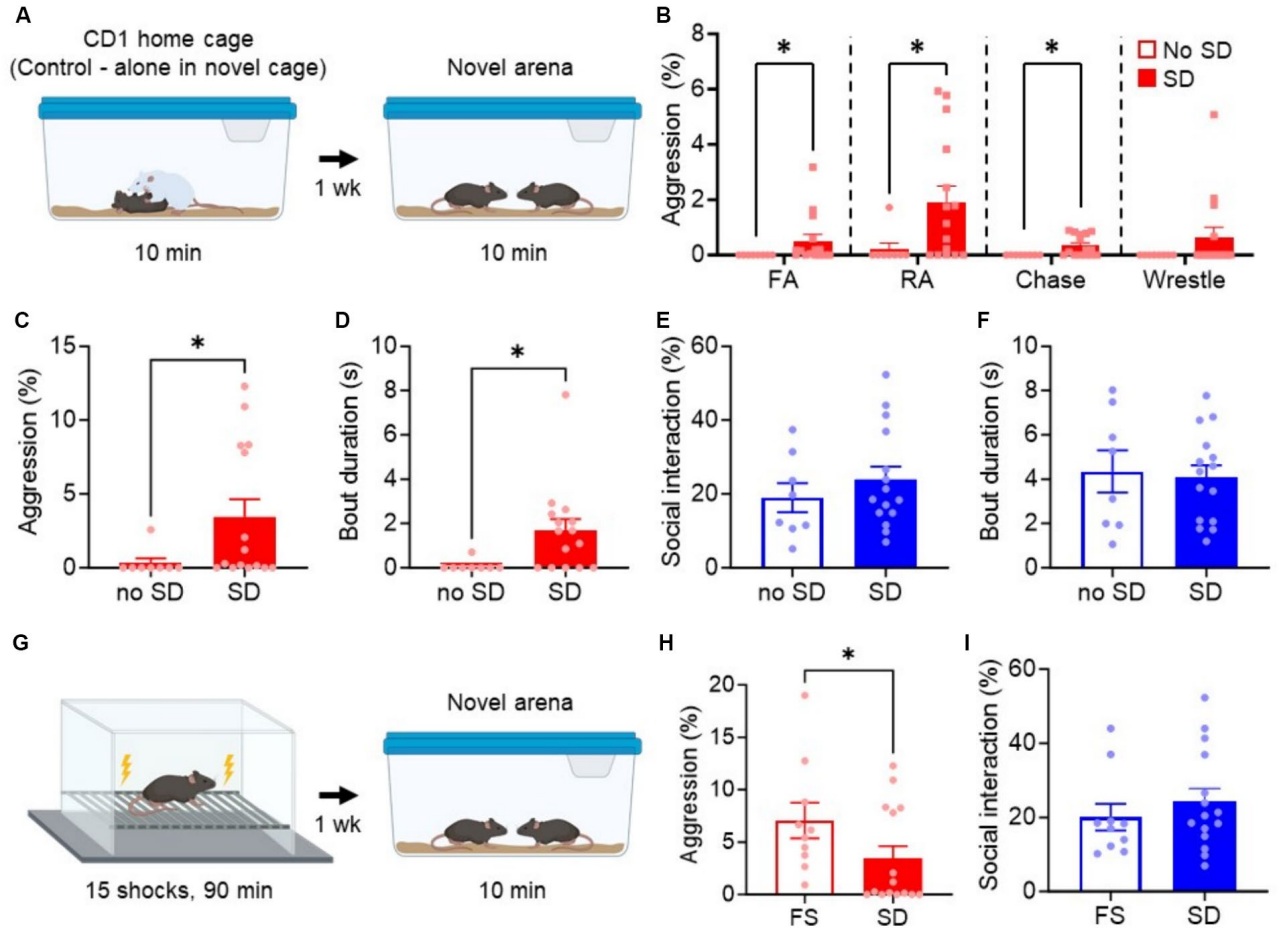
Acute social defeat stress promotes long-lasting aggression, but not non-aggressive social interaction. **(A)** Experimental paradigm for testing the effect of SD on long-lasting aggression in a novel arena. Animals in the cage without exposure to SD were used as controls (no SD). **(B)** Breakdown of individual aggressive behaviors during the aggression test. FA, Front attack; RA, Rear attack. **(C–F)** Quantification of attack time **(C)**, attack bout duration **(D)**, non-aggressive social behavior **(E)**, and non-aggressive social bout duration **(F)** during aggression test. **(G)** Experimental paradigm for foot shock (FS)-induced aggression. **(H,I)** Comparison of FS and SD-induced aggression **(H)** or non-aggressive social behavior **(I)**. Data are mean ± SEM. ^*^p = 0.05.

Following the 4 weeks isolation period, we moved the mice to a novel arena either alone (control condition) or containing a larger CD1 mouse, a strain known for its aggressive behavior relative to the C57 strain (Golden et al., 2011). This acute exposure lasted for a controlled duration of 10 min. One week after the SD event, we conducted aggression tests on the defeated mice or controls (Figure 1A).

The mice subjected to the SD paradigm exhibited a significant increase in aggressive behavior and bout duration compared to their non-attacked counterparts (no SD, *p* = 0.023, Figure 1C; *p* = 0.037, Figure 1D). An analysis of the individual aggressive behaviors performed during the aggression test revealed a significant increase in rear attacks, front attacks, and chasing behavior [Figure 1B; Front attacks (FA), *p* = 0.011; Rear attacks (RA), *p* = 0.016; Chasing, *p* = 0.017; Wrestling, *p* = 0.257]. Front attacks, such as those to the face, neck, and belly, are common indicators of excessive or escalated aggression (Miczek et al., 2013), and so it is notable that SD mice perform these behaviors, which are consistent with our previous findings with foot shock-induced aggression (Nordman J. et al., 2020; Nordman J. C. et al., 2020). Interestingly, non-aggressive social interaction and bout duration were not different between groups (*p* = 0.428, Figure 1E; *p* = 0.806, Figure 1F), also consistent with our previous studies. No correlation was found between the amount of aggression and non-aggressive social interaction in the SD group (*r* = 0.2008, *p* = 0.358). These results suggest that SD specifically heightens aggressive responses in a novel arena without broadly altering other aspects of social interaction.

Finally, we compared SD-induced aggression to foot shock-induced aggression, our previous model for promoting long-lasting attack behavior after traumatic stress. We found that foot shocked mice were overall more aggressive than SD mice (*p* = 0.047, Figure 1H), but social behavior remained the same (*p* = 0.338, Figure 1I). These results suggest that SD and foot shock-induced aggression are qualitatively similar but quantitatively different, supporting the use of either stressor in studying the impact of stress on aggression.

### 3.2 Acute social defeat activates the MeApv and VmHvl

Our previous studies demonstrated that an MeApv-VmHvl pathway drives foot shock-induced aggression (Nordman J. et al., 2020; Nordman J. C. et al., 2020). To test if the MeApv underlies SD-induced aggression, we first analyzed whether the cells in the MeApv are activated following SD.

**Figure 2:**
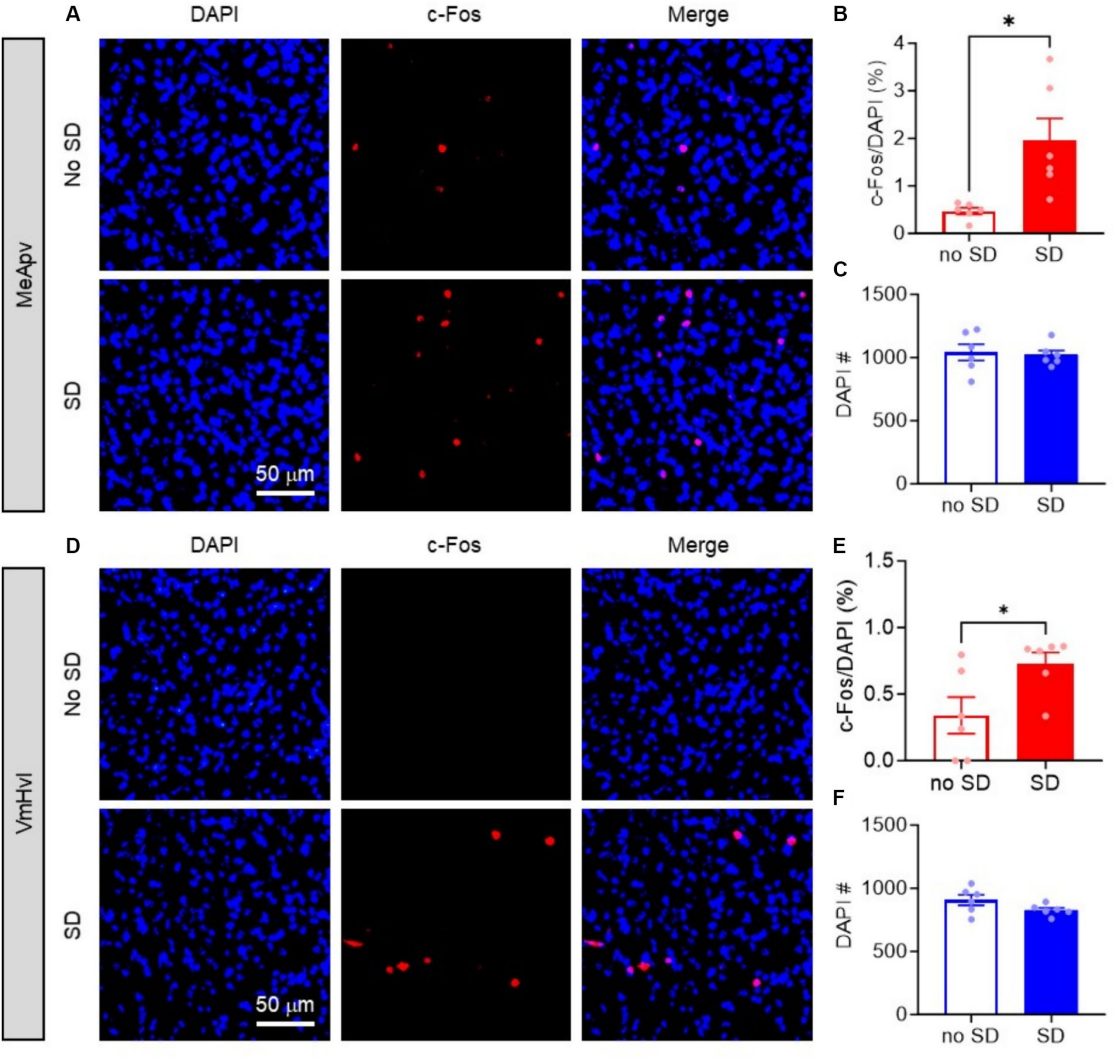
Acute social defeat activates the MeApv and VmHvl. **(A,D)** Representative images of c-Fos and DAPI labeled cells in the MeApv **(A)** and VmHvl **(D)** 30 min after SD or control condition (No SD). **(B)** Percentage of c-Fos^+^ cells **(B,E)** or number of DAPI^+^ cells **(C,F)** in the MeApv and VmHvl from mice exposed to SD or no SD. Data are mean ± SEM. ^*^p = 0.05.

To begin, mice were exposed to SD and then euthanized for immunohistochemical analysis 30 min later. Tissue slices containing the MeApv were immunostained for the immediate early gene marker c-Fos, a widely used marker to detect neural activation (Morgan et al., 1987). Imaging results demonstrated a significant increase in c-Fos expression within the MeApv in SD animals compared to no SD controls (*p* = 0.011; Figures 2A,B). No differences were found in the number of DAPI cells between conditions (*p* = 0.791; Figure 2C). These results suggest that SD increases aggression through activation of the MeApv.

We also examined whether the VmHvl, the downstream target of the MeApv, which we have shown to be critical for foot shock-induced aggression, was also activated by SD. We analyzed c-Fos expression in the same tissue as above. As expected, we found significantly more c-Fos + cells in the VmHvl of SD mice compared to no SD controls (*p* = 0.026, Figures 2D,E). Again, no differences were found in the number of DAPI cells between conditions (*p* = 0.118, Figures 2D,F). These results suggest the involvement of the MeApv-VmHvl pathway in SD-induced aggression.

### 3.3 Chemogenetic inhibition of excitatory neurons in the MeApv decreases acute social defeat-induced aggression

**Figure 3:**
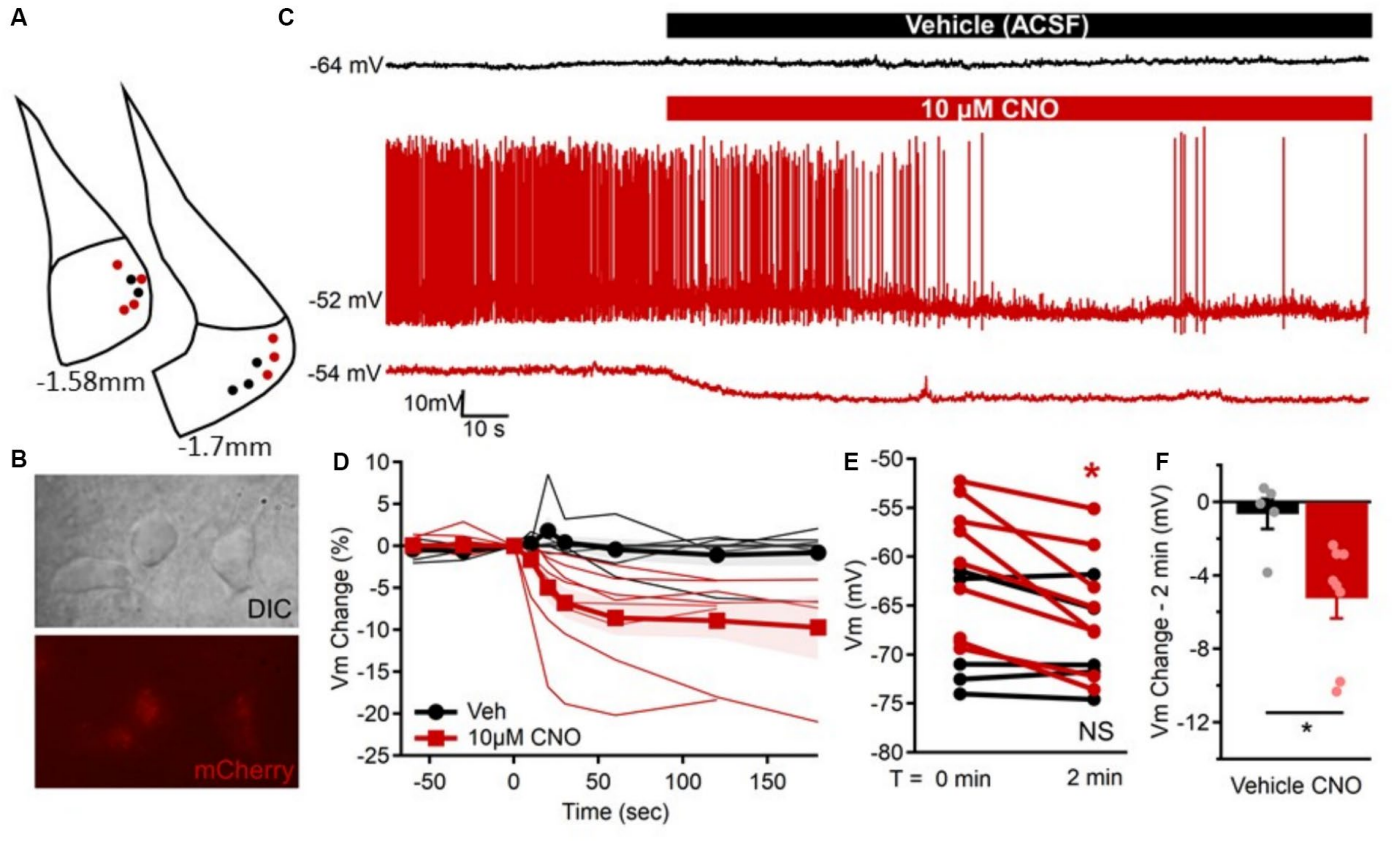
Confirmation that CNO inhibits hM4D(Gi)-expressing excitatory MeApv neurons. **(A)** Location of mCherry-positive MeApv neurons recorded from in response to “vehicle” (black) or 10 μM clozapine-n-oxide (CNO, red). **(B)** Differential interference contrast (DIC, top) and corresponding mCherry expression (bottom) of neurons targeted for recording in the MeApv. **(C)** Example membrane potential recordings of one mCherry-positive MeApv neuron in response to “vehicle” (ACSF, black) and two other neurons in response to 10 μM CNO (red). Typically, neurons were not spontaneously active, but CNO application in the bath ACSF was sufficient to reduce firing and cause hyperpolarization of the membrane potential similar to that observed in quiescent neurons. **(D)** The percent change in the membrane potential for all neurons (thin lines) and group mean ± SEM (thick lines with marker) prior to and up to 3 min after “vehicle” (black, circles) or CNO (red, squares) application. **(E)** Comparison of the resting membrane potential for all cells immediately before (T = 0 min) and 2 min after exposure to “vehicle” (black, *p* = 0.476; *n* = 5) or 10 μM CNO (red, *p* = 0.002; *n* = 8) when the change in membrane potential typically stabilized. **(F)** Individual neuron and average group changes in membrane potential at 2 min after 10 μM CNO application (*p* = 0.018). Data are individual observations or mean ± SEM as indicated. ^*^p = 0.05.

**Figure 4:**
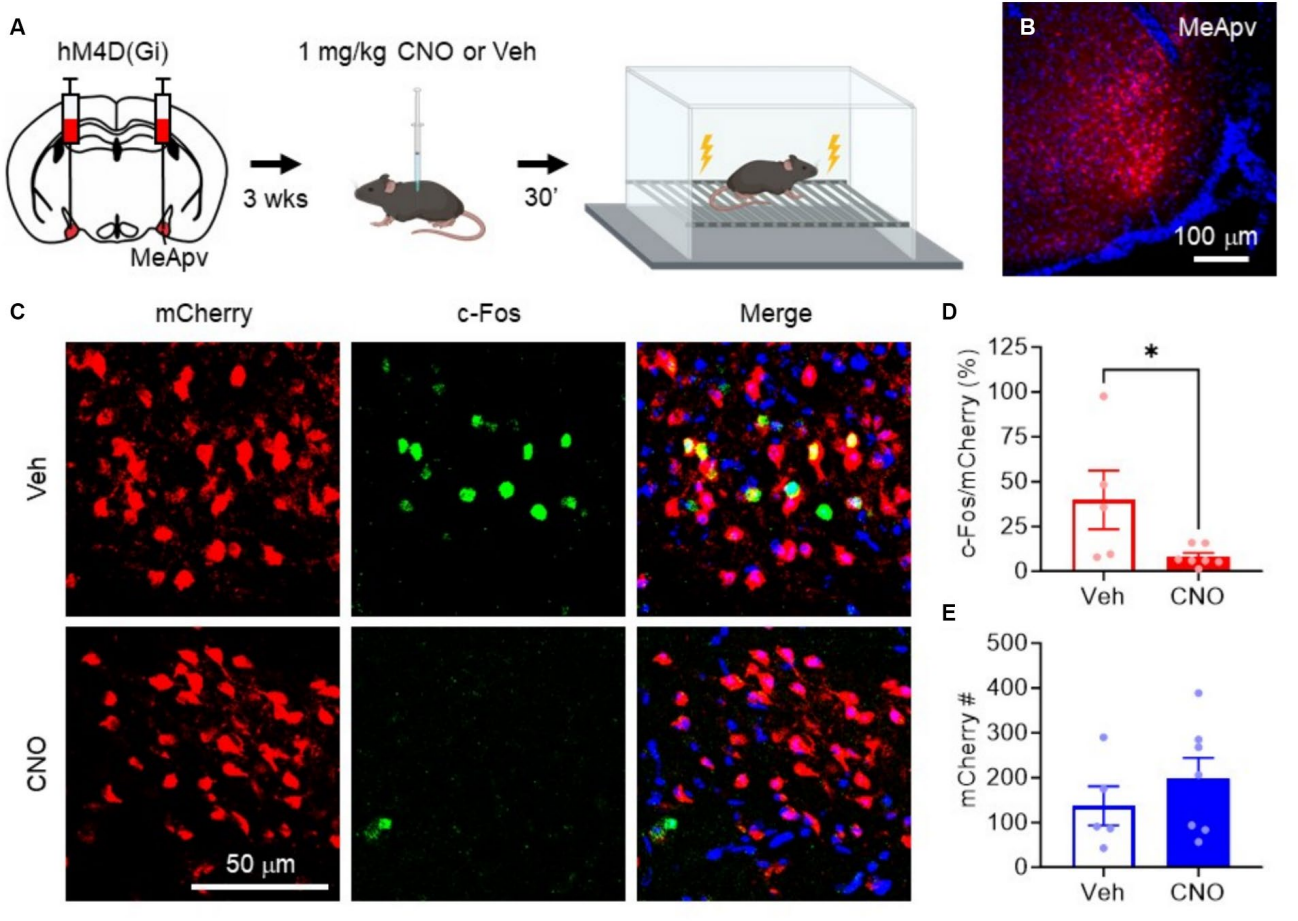
hM4D(Gi) inhibits excitatory MeApv neuronal activation by foot shock. **(A)** Experimental procedure. Mice were injected with hM4D(Gi) under the control of the CaMKIIa promoter. 3 weeks later, the mice were injected with 1 mg/kg CNO or vehicle 30 min before non-contingent foot shock. Mice were then sacrificed for c-Fos immunolabeling. **(B)** Representative image of the hM4D(Gi)-expressing neurons in the MeApv. **(C)** Representative images of MeApv neurons expressing mCherry, c-Fos, or both, 30 min after foot shock in mice treated with CNO or Vehicle, described in panel **(A)**. **(D)** Percentage of mCherry+ neurons that colocalize with c-Fos between conditions. **(E)** Number of mCherry+ neurons between conditions. Data are mean ± SEM. ^*^p = 0.05.

We previously found that excitatory neurons in the MeApv drive foot shock-induced aggression (Nordman J. et al., 2020; Nordman J. C. et al., 2020). To test the role of the excitatory MeApv neurons in SD-induced aggression, we used a chemogenetic approach. We began by bilaterally injecting the Designer Receptors Exclusively Activated by Designer Drugs (DREADD) virus hM4D(Gi) or mCherry control into the MeApv of 4–5-week-old mice (Figures 3A, 4A). hM4D(Gi) as an inhibitory DREADD receptor that chemogenetically inhibits neurons (Roth, 2016). Both the hM4D(Gi) and mCherry control viruses were under the control of the calcium–calmodulin-dependent protein kinase II alpha (CaMKIIα) promoter to restrict expression of our transgene to excitatory MeApv neurons (Nordman J. C. et al., 2020). Mice were then socially isolated for 3 weeks.

To confirm that the hM4D(Gi) receptor inhibits excitatory MeApv neurons, we performed *ex vivo* electrophysiological recordings using the hM4D(Gi)-specific synthetic ligand clozapine N-oxide (CNO) (Chang and Gean, 2019). We began by recording the membrane potential from mCherry-positive neurons in the MeApv (Figures 3A,B) with comparable membrane capacitance (44.9 ± 3.5 pF), resistance (320 ± 29.2 MΩ), and resting potential (−63.6 ± 1.9 mV). Although these neurons were not typically spontaneously active (Figure 3C), the membrane potentials were stable overtime and comparison of membrane potentials before and after a pseudo-‘vehicle’ application indicated they were not different (*p* = 0.476, Figure 3E). However, over a similar timeframe, the bath application of CNO (10 μM) rapidly, reliably, and significantly hyperpolarized the membrane potential in all neurons evaluated (*p* = 0.002, Figures 3C–E) with an effect that typically stabilized after 2–3 min of CNO exposure (Figure 3D). Bath application of CNO induced membrane hyperpolarization by 4–18 mV within 2 min (−8.0 ± 2 mV) which was significantly different (*p* = 0.018) from the change observed in ‘vehicle’ exposed neurons (−1.1 ± 1.33 mV, Figure 3F).

To confirm that the hM4D(Gi) receptor can inhibit excitatory MeApv neurons *in vivo*, we performed c-Fos staining after foot shock. We chose foot shock as it is a more reliable stressor than SD due to the variability of CD1 attacks and because we have consistently shown that foot shock activates MeApv neurons *in vivo*. We began by administering intraperitoneal (IP) injections of 1 mg/kg CNO, the effective dose in our previous study (Bartsch et al., 2023b), or vehicle 30 min before non-contingent foot shock (Figures 4A,B). The mice were then euthanized 30 min later for c-Fos immunolabeling. CNO-treated mice had significantly fewer c-Fos + MeApv neurons that co-localized with hM4D(Gi)-mCherry compared to vehicle-treated mice (*p* = 0.044, Figures 4C,D), but had the same number of mCherry+ MeApv neurons between conditions (*p* = 0.389, Figures 4C,E). These results confirm the efficacy of hM4D(Gi) and CNO in inhibiting the excitatory MeApv neurons during stress *in vivo*.

**Figure 5:**
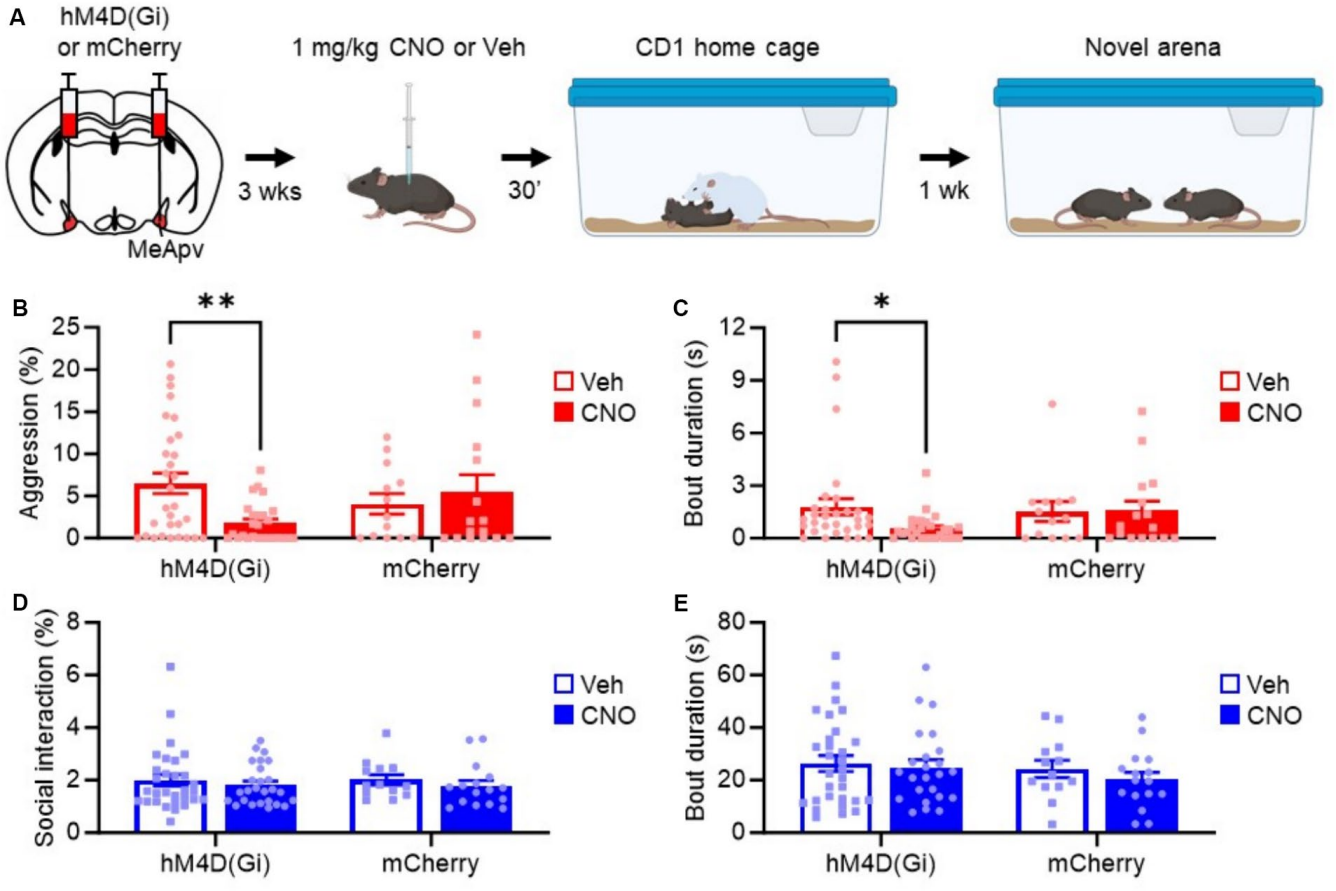
Chemogenetic inhibition of excitatory MeApv neurons suppresses acute social defeat-induced aggression. **(A)** Experimental schedule. Mice received bilateral injections of hM4D(Gi) or mCherry control virus into the MeApv, followed 3 weeks later by IP injections of 1 mg/kg CNO or Veh 30 min before SD. Aggression was tested 1 week later. **(B–E)** Quantification of attack time **(B)**, attack bout duration **(C)**, non-aggressive social behavior **(D)**, and non-aggressive social bout duration **(E)** 7 days after SD for all conditions, described in panel **(A)**. Mean ± SEM, ^*^p < 0.05, ^**^p < 0.01.

To assess the role of the MeApv neurons in SD-induced aggression, we delivered IP injections of 1 mg/kg CNO or vehicle 30 min before the SD test (Figure 5A). Aggression testing was performed 1 week later. Our behavioral analysis showed a decrease in attack behavior in mice expressing hM4D(Gi) that received CNO as compared to vehicle-treated mice or mice expressing mCherry control and treated with CNO or vehicle [*F*(1, 81) = 5.697, *p* = 0.019, Figure 5B; *F*(1, 83) = 2.010, *p* = 0.160, Figure 5C]. No change in non-aggressive social interaction or bout duration was observed [*F*(1, 79) = 0.1555, *p* = 0.694, Figure 5D; *F*(1, 79) = 0.155, p = 0.694, Figure 5E]. These results strongly suggest that the excitatory neurons of the MeApv mediate SD-induced long-lasting aggression.

## 4 Discussion

### 4.1 Summary of findings

In this study, we investigated the neurobiological underpinnings of aggression following SD during adolescence, with a focus on the MeApv. We demonstrated that SD not only promotes long-lasting aggression in mice but also significantly activates the MeApv and VmHvl, critical members of the aggression circuit that drives foot shock-induced aggression. Importantly, when we inhibited excitatory neurons within the MeApv using chemogenetic tools, we observed a marked reduction in aggression. These findings emphasize the critical role of the MeApv and suggest the involvement of the MeApv-VmHvl pathway in mediating aggression following traumatic social experiences and present it as a potential target for clinical interventions.

### 4.2 The MeApv and VmHvl in traumatic stress-induced aggression

Our findings suggest that SD acts as a potent stressor that triggers aggression through specific neural circuits, notably the MeApv. Unlike foot shock, which is a non-social stressor and might not fully capture the complexity of human traumatic experiences, SD mimics the interpersonal conflicts relevant to human social stress. This relevance is critical as it enhances the ecological validity of the model, providing a more accurate representation of how traumatic experiences can lead to aggression in humans.

The activation of the MeApv and VmHvl immediately after SD and foot shock and the efficacy of MeApv inhibition in suppressing the ensuing long-lasting aggression underscores the importance of these regions in stress-induced aggression. While activation of these regions after traumatic stress is transient, our previous research has demonstrated that foot shock promotes long-lasting aggression by strengthening synaptic connections in an excitatory MeApv-VmHvl pathway (Nordman J. et al., 2020; Nordman J. C. et al., 2020; Bartsch et al., 2023b). These alterations were shown to persist for a week at testing, and likely longer. The current study extends these findings by suggesting that similar mechanisms may be at play following SD, which also calls for examining whether this stressor induces comparable synaptic and structural changes in the MeApv-VmHvl pathway. These findings would provide a general account of the role of the MeApv-VmHvl pathway in aggression escalation brought on by traumatic stress.

### 4.3 Problems, potential pitfalls, and future directions

While our results are robust, the methods employed, such as c-Fos staining and chemogenetic manipulations, present limitations primarily due to their temporal resolution. These techniques provide insights into MeApv activity and function only shortly after SD, but do not capture the immediate neural dynamics during the CD1 encounters. Moreover, c-Fos does not allow us to monitor more persistent changes that may occur. To overcome this, future studies could implement real-time techniques such as fiber photometry or *in vivo* electrophysiology, which would allow for a moment-to-moment analysis of neural circuitry during aggressive behaviors over the short- and long-term.

Another limitation is the lack of consideration for social context, particularly the dominance status and defeat resistance of the animals. For example, stress-resilient mice are less sensitive to the adverse effects of chronic social defeat in most metrics of anxiety-like, depression-like, and social behavior (Krishnan et al., 2007). Interestingly, while dominant and subordinate mice have similar anxiety-like and depression-like profiles after chronic social defeat, dominant mice appear to display greater social deficits as measured by social avoidance (Larrieu et al., 2017). Furthermore, dominant mice show heightened activity in the MeA after social defeat stress (Cooper et al., 2023). These findings could explain the variability in aggression between the defeated mice in our study (Figure 1C), as stress resilience and dominance status were not controlled for. It is also notable that foot shock produces a significantly stronger effect on aggression, which is less variable, compared to SD (Figure 1H), also plausibly attributable to the sensitivity of the mice to social stress and social status.

Interestingly, SD had no effect on social behavior (Figures 1E,F) and chemogenetic inhibition of the MeApv did not alter social interaction or bout duration. These results suggest one of two possibilities given the studies outlined above: chronic and acute social defeat produce different effects on social behavior, or less likely, the mice used in our study were generally more subordinate, buffering them against the social deficits observed in other studies (Cooper et al., 2023). Nevertheless, future research should incorporate social variables like stress sensitivity and dominance status to fully elucidate their impact on maladaptive behaviors induced by SD.

While we did not test the effect of SD on female aggression, this is an important consideration and worthy of examination in future studies. Findings would either reveal that traumatic stress promotes aggression in males and females, showing this to be a generalizable phenomenon, or, if no aggression increase is observed after SD, that there are important sex differences in the susceptibility of the aggression circuitry to stress.

Finally, while this study focused primarily on aggression, other behavioral dimensions such as anxiety, depression, or fear responses to SD were not examined but could be intricately linked to the aggression pathways (Golden et al., 2011). However, as we show in our previous studies, these traumatic stress-induced behavioral effects are not altered after manipulating the MeApv neurons or the MeApv-VmHvl pathway, and so are unlikely to be affected after SD as well. That said, exploring these behavioral dimensions would provide a broader understanding of the psychopathological consequences of traumatic stress, and SD specifically.

### 4.4 Conclusion and clinical implications

This study solidifies the role of the MeApv in the onset of aggression following SD, highlighting its function in the neural circuitry mediating trauma-induced behavioral responses. The modulation of this pathway, particularly through the inhibition of excitatory MeApv neurons, offers a promising avenue for therapeutic intervention.

Our previous research has also demonstrated that traumatic stress-induced aggression can be ameliorated using N-methyl-D-aspartate receptor (NMDAR) antagonists that likely operate at MeApv-VmHvl synapses (Nordman J. et al., 2020; Nordman J. C. et al., 2020; Nordman et al., 2022; Bartsch et al., 2023a). Therefore, pharmacologically targeting NMDARs could be pivotal in developing treatments aimed at mitigating the adverse effects of traumatic stress (Nordman, 2021; Bartsch and Nordman, 2022), and should be explored in relation to SD-induced aggression.

Our findings not only advance the understanding of the neural basis of aggression but also underscore the potential for developing targeted interventions that could prevent the long-term behavioral consequences of traumatic experiences. Such therapeutic strategies hold promise for interrupting the cycle of violence that traumatic stress can perpetuate, providing a foundation for more effective treatments in clinical settings.

## Data availability statement

The original contributions presented in the study are included in the article/supplementary material; further inquiries can be directed to the corresponding author.

## Ethics statement

The animal study was approved by Animal Care and Use Committee of Southern Illinois University School of Medicine. The study was conducted in accordance with the local legislation and institutional requirements.

## Author contributions

NM: Data curation, Formal analysis, Investigation, Methodology, Validation, Visualization, Writing – original draft, Writing – review & editing. MA: Data curation, Formal analysis, Investigation, Methodology, Validation, Visualization, Writing – original draft, Writing – review & editing. EQ: Formal analysis, Investigation, Methodology, Validation, Visualization, Writing – review & editing. SA: Data curation, Investigation, Methodology, Validation, Visualization, Writing – review & editing. TA: Formal analysis, Investigation, Methodology, Validation, Visualization, Writing – review & editing. JJ: Formal analysis, Investigation, Methodology, Validation, Visualization, Writing – review & editing. BR: Data curation, Formal analysis, Funding acquisition, Investigation, Methodology, Project administration, Resources, Software, Supervision, Validation, Visualization, Writing – original draft, Writing – review & editing. JN: Conceptualization, Formal analysis, Funding acquisition, Investigation, Methodology, Project administration, Resources, Software, Supervision, Validation, Visualization, Writing – original draft, Writing – review & editing.
